# Power and Stability Properties of Resampling-Based Multiple Testing Procedures with Applications to Gene Oncology Studies

**DOI:** 10.1155/2013/610297

**Published:** 2013-11-20

**Authors:** Dongmei Li, Timothy D. Dye

**Affiliations:** ^1^Department of Public Health Sciences, Office of Public Health Studies, The University of Hawaii at Manoa, 1960 East-West Road, Honolulu, HI 96822, USA; ^2^Department of Obstetrics, Gynecology, and Women's Health, John A. Burns School of Medicine, University of Hawaii, 651 Ilalo Street, Honolulu, HI 96813, USA

## Abstract

Resampling-based multiple testing procedures are widely used in genomic studies to identify differentially expressed genes and to conduct genome-wide association studies. However, the power and stability properties of these popular resampling-based multiple testing procedures have not been extensively evaluated. Our study focuses on investigating the power and stability of seven resampling-based multiple testing procedures frequently used in high-throughput data analysis for small sample size data through simulations and gene oncology examples. The bootstrap single-step min*P* procedure and the bootstrap step-down min*P* procedure perform the best among all tested procedures, when sample size is as small as 3 in each group and either familywise error rate or false discovery rate control is desired. When sample size increases to 12 and false discovery rate control is desired, the permutation max*T* procedure and the permutation min*P* procedure perform best. Our results provide guidance for high-throughput data analysis when sample size is small.

## 1. Introduction

With rapidly developing biotechnology, microarrays and next generation sequencing have been widely used in biomedical and biological fields for identifying differentially expressed genes, detecting transcription factor binding sites, and mapping complex traits using single nucleotide polymorphisms (SNPs) [1–7]. The multiple testing error rates associated with thousands, even millions of hypotheses testing, need to be taken into account. Common multiple testing error rates controlled in multiple hypotheses testing are the familywise error rate (FWER), which is the probability of at least one false rejection [8, 9] and the false discovery rate (FDR), which is the expected proportion of falsely rejected null hypotheses [10].

Resampling-based multiple testing procedures are widely used in high-throughput data analysis (e.g., microarray and next generation sequencing), especially when the sample size is small or the distribution of test statistic is nonnormally distributed or is unknown. Resampling-based multiple testing procedures can account for dependent structures among *P*  values or test statistics, resulting in lower type II errors. The commonly used resampling techniques include permutation tests and bootstrap methods. 

Permutation tests are nonparametric statistical significance tests, where the test statistics' distribution under the null hypothesis is constructed by calculating all possible values or a concrete number of test statistics (usually 1000 or above) from permuted observations under the null hypothesis. The theory of the permutation test is based on the work by Fisher and Pitman in the 1930s. Permutation tests are distribution-free, which can provide exact *P*  values even when sample size is small. 

The bootstrap method, first introduced by Efron [11] and further discussed by Efron and Tibshirani [12], is a way of approximating the sampling distribution from just one sample. Instead of taking many simple random samples from the population to find the sampling distribution of a sample statistic, the bootstrap method repeatedly samples with replacement from one random sample. Efron [11] showed that the bootstrap method provides an asymptotically unbiased estimator for the variance of a sample median and for error rates in a linear discrimination problem (outperforming cross-validation). Freedman [13] conclusively showed that bootstrap approximation of the distribution of least square estimates is valid. Finally, Hall [14] showed that the bootstrap method's reduction of error coverage probability, from *o*(*n* ^−1/2^) to *o*(*n* ^−1^), makes the bootstrap method one order of magnitude more accurate than the delta method. The *P*  values computed by the bootstrap method are less exact than *P*  values obtained from the permutation method, and additionally, *P*  values estimated by the bootstrap method are asymptotically convergent to the true *P*  values [15]. 

Different resampling methods can draw different conclusions, however, when applied to the same data set. An investigation of multiple testing error rate control, power, and stability of those resampling methods under different situations is necessary to provide guidance for data analysis, so that optimal methods in different scenarios could be used to maximize power and minimize multiple testing error rates.

In this paper, we focus on investigating the power and stability properties of several commonly used resampling-based multiple testing procedures: (1) the permutation tests [16]; (2) the permutation-based significant analysis of microarray (SAM) procedure [17]; and (3) the bootstrap multiple testing procedures [15]. 

## 2. Materials and Methods

### 2.1. Permutation Test

To carry out a permutation test based on a test statistic that measures the size of an effect of interest, we proceed as follows.

1. Compute the test statistics for the observed data set, such as two sample *t*-test statistics.
2. Permute the original data in a way that matches the null hypothesis to get permuted resamples and construct the reference distribution using the test statistics calculated from permuted resamples.
3. Calculate the critical value of a level *α* test based on the upper *α* percentile of the reference distribution, or obtain the raw *P* value by computing the proportion of permutation test statistics that are as extreme as or more extreme than the observed test statistic.

Westfall and Young [16] proposed two methods to adjust raw *P*  values to control the multiple testing error rates. One is single-step min*P* procedure and the other is single-step max*T* procedure.

The single-step min*P* adjusted *P*  values are defined as [18]

(1) $$\begin{array}{c} {\tilde{p}}_{i}=\mathrm{Pr}\left(\underset{1\leq l\leq m}{\min}P_{l}\leq p_{i}\;|\;H_{M}\right). \end{array}$$

The single-step max*T* adjusted *P*  values are defined in terms of test statistics *T*_*i*_, namely, [18]

(2) $$\begin{array}{c} {{\tilde{p}}_{i}}^{T}=\mathrm{Pr}\left(\underset{1\leq l\leq m}{\max}\left|T_{l}\right|\geq \left|t_{i}\right|\;|\;H_{M}\right), \end{array}$$

where *H*_*M*_ is the complete null hypothesis. *P*_*l*_ is the raw *P* value for the *l*th hypothesis, and *T*_*l*_ is the observed test statistic for the *l*th hypothesis.

### 2.2. Significance Analysis of Microarrays (SAM) Procedure

The Significance Analysis of Microarrays (SAM) procedure proposed by Tusher et al. [17] identifies genes with significant changes in expression using a set of gene-specific *t*-tests. In SAM, genes are assigned with scores relative to change in gene expression and its standard deviation of repeated measurements. Scatter plots of the observed relative differences and the expected relative differences estimated through permutation identifies statistically significant genes based on a fixed threshold.

Based on the description of SAM in Tusher et al. [17], the SAM procedure can be summarized as follows.

1. Compute a test statistic *t*_*i*_ for each gene *i*  (*i* = 1,…, *g*).
2. Compute order statistics *t*_(*i*)_ such that *t*_(1)_ ≤ *t*_(2)_ ≤ ⋯≤*t*_(*g*)_.
3. Perform *B* permutations of the responses/covariates *y*_1_,…, *y*_*n*_. For each permutation *b*, compute the permuted test statistics *t*_*i*,*b*_ and the corresponding order statistics *t*_(1),*b*_ ≤ *t*_(2),*b*_ ≤ ⋯≤*t*_(*g*),*b*_.
4. From the *B* permutations, estimate the expected values of order statistics by ${\overset{-}{t}}_{\left(i\right)}=(1/B)\sum_{b=1}^{B}t_{(i),b}$.
5. Form a quantile-quantile (Q-Q) plot (SAM plot) of the observed *t*_(*i*)_ versus the expected ${\overset{-}{t}}_{\left(i\right)}$.
6. For a given threshold Δ, starting at the origin, and moving up to find the first *i* = *i*_1_ such that $t_{\left(i\right)}-{\overset{-}{t}}_{\left(i\right)}>\Delta$. All genes past *i*_1_ are called significant positives. Similarly, starting at the origin and moving down to the left, find the first *i* = *i*_2_ such that ${\overset{-}{t}}_{\left(i\right)}-t_{\left(i\right)}>\Delta$. All genes past *i*_2_ are called significant negatives. Define the upper cut point Cut_up_(Δ) = min⁡⁡{*t*_(*i*)_ : *i* ≤ *i*_1_} = *t*_(*i*_1_)_ and the lower cut point Cut_low_(Δ) = max⁡⁡{*t*_(*i*)_ : *i* ≥ *i*_2_} = *t*_(*i*_2_)_.
7. For a given threshold, the expected number of false rejections *E*(*V*) is estimated by computing the number of genes with *t*_*i*,*b*_ above Cut_up_(Δ) or below Cut_low_(Δ) for each of the *B* permutation and averaging the numbers over *B* permutations.
8. A threshold Δ is chosen to control the Fdr  (Fdr = (*E*(*V*)/*r*)) under the complete null hypothesis, at an acceptable nominal level.

### 2.3. Bootstrap Method

The bootstrap method based on estimated null distribution of test statistics was introduced by Pollard and van der Laan [15] and proceeds as follows:

1. Compute the observed test statistic for the observed data set.
2. Resample the data with replacement within each group to obtain bootstrap resamples, compute the resampled test statistics for each resampled data set, and construct the reference distribution using the centered and/or scaled resampled test statistics.
3. Calculate the critical value of a level *α* test based on the upper *α* percentile of the reference distribution, or obtain the raw *P*  values by computing the proportion of bootstrapped test statistics that is as extreme as or more extreme than the observed test statistic.

The MTP function based on the bootstrap method includes single-step min*P* and max*T* adjusted *P*  values, as well as step-down min*P* and step-down max*T* adjusted *P*  values. The single-step max*T* and min*P* adjusted *P*  values are defined as before. 

The step-down min*P* adjusted *P*  values are defined as

(3) $$\begin{array}{c} {\tilde{p}}_{r_{i}}=\underset{k=1,\ldots ,i}{\max}\left\{\text{Pr}\left(\underset{l=k,\ldots ,m}{\min}P_{r_{l}}\leq p_{r_{k}}\;|\;H_{M}\right)\right\}, \end{array}$$

and the step-down max*T* adjusted *P*  values are defined as

(4) $$\begin{array}{c} {\tilde{p}}_{S_{i}}=\underset{k=1,\ldots ,i}{\max}\left\{\text{Pr}\left(\underset{l=k,\ldots ,m}{\max}\left|T_{s_{l}}\right|\geq \left|t_{s_{k}}\right|\;|\;H_{M}\right)\right\}, \end{array}$$

where |*t*_*s*_1__ | ≥|*t*_*s*_2__ | ≥⋯≥|*t*_*s*_*m*__| denote the ordered test statistics [18].

### 2.4. Simulation Setup

Simulation studies were conducted to compare the power and stability of the resampling-based multiple testing procedures for both independent test statistics and dependent test statistics. According to Rubin et al. [19], the power is defined as the expected proportion of true positives. The stability is measured as the variance of true discoveries and variance of total discoveries. 

In our first simulation study, each set includes 100 independently generated groups of two samples with equal sample size of 3 or 12 in each group. 100 repetitions are chosen because computationally 100 is more efficient than 1000 or even higher repetitions. 1000 repetitions are also tried and similar results are obtained. Thus, 100 repetitions are chosen for computational efficiency. The total number of genes (*m*) is set to be 2000 with the fraction of true null hypotheses (*m*_0_/*m*) at 50%. In the two-group comparison, the standardized logarithms of gene expression levels are generated from multivariate normal distribution. One group has 50% of genes with means at *μ* and the remaining with means at 0. All genes in the other group have means at 0. The mean expression level *μ* on log2 scale is set to be from 1 to 6 with step 0.50 for the first simulation study. The variances of the standardized logarithm of gene expression levels are equal to 1 in both groups. Thus, the mean differences of *μ* in gene expression between the two groups are the Cohen's *d* effect sizes. The pairwise correlation coefficients of test statistics are set to be 0 in our simulation study. The test statistics used are equal variance *t*-test throughout the simulation study. The FWER/FDR level is set at 5% (*α* = 0.05).

We conducted another simulation study to examine the effect of fraction of true null hypotheses on power and stability. In our second simulation study, each data set includes 100 independently generated samples of two groups with equal sample size of 3. The total number of genes (*m*) is set to be 1000, with the fraction of differentially expressed genes ((*m* − *m*_0_)/*m*) equal to 10%, 25%, 50%, 75%, and 90% to cover all possible scenarios. In the two-group comparison, the gene expression level on log2 scale is generated randomly from a multivariate normal distribution with *μ* = 0 and *σ* = 1. The correlations between genes are randomly fluctuated between 0 and 1 to mimic the correlations in real microarray data. The mean differences are set between 1 and 2, with the step equaling the inverse of the number of differentially expressed gene (*m* − *m*_0_). The variances are set to 1. Equal variance *t*-tests are used for this simulation study, and the FWER/FDR level is set at 5% (*α* = 0.05).

The mt.max*T* and mt.min*P* functions in *R* were used to evaluate the Westfall and Young's permutation test. The sam function in *R* was used for the SAM procedure. The Bootstrap method proposed by Pollard and van der Laan [15] was executed using the MTP function in *R*. The MTP function includes the max*T* method, the min*P* method, the single step procedure, and the step-down procedure, which results into four different functions, including single-step max*T* (ss.max*T*), single-step min*P* (ss.min*P*), step-down max*T* (sd.max*T*), and step-down min*P* (sd.min*P*).

### 2.5. Cancer Microarray Example

Ovarian cancer is a common cause of cancer deaths in women [20]. Microarray experiments were conducted to identify differentially expressed genes between chemotherapy favorable patients and chemotherapy unfavorable patients [21]. Those differentially expressed genes could be used to develop optimal treatment for a new ovarian cancer patient by predicting possible response to chemotherapy. The gene expression data of 12,625 genes from 6 patients' mRNA samples, obtained from Moreno et al.'s ovarian cancer microarray study, were used to show the differences in the number of total discoveries among those resampling-based multiple testing procedures with FWER or FDR controlled at 5% (data accessible at NCBI GEO database [22], accession GSE7463). The preprocessing of the ovarian cancer data set was done using the RMA background correction, quantile normalization, and robust linear model summarization. The raw *P*  values and the adjusted *P*  values of comparisons between the chemotherapy favorable group (3 subjects) and chemotherapy unfavorable group (3 subjects) were calculated using the resampling-based multiple testing functions in the multitest package and the siggenes package in *R*. 

## 3. Results

Simulation studies were conducted to compare the power and stability across all tested multiple testing procedures for normally distributed data with either independent or randomly correlated test statistics. The sample size is 3 or 12 in each group for independent test statistics and 3 in each group for randomly correlated test statistics.

### 3.1. Simulation Results for Independent Test Statistics

**Figure 1:**
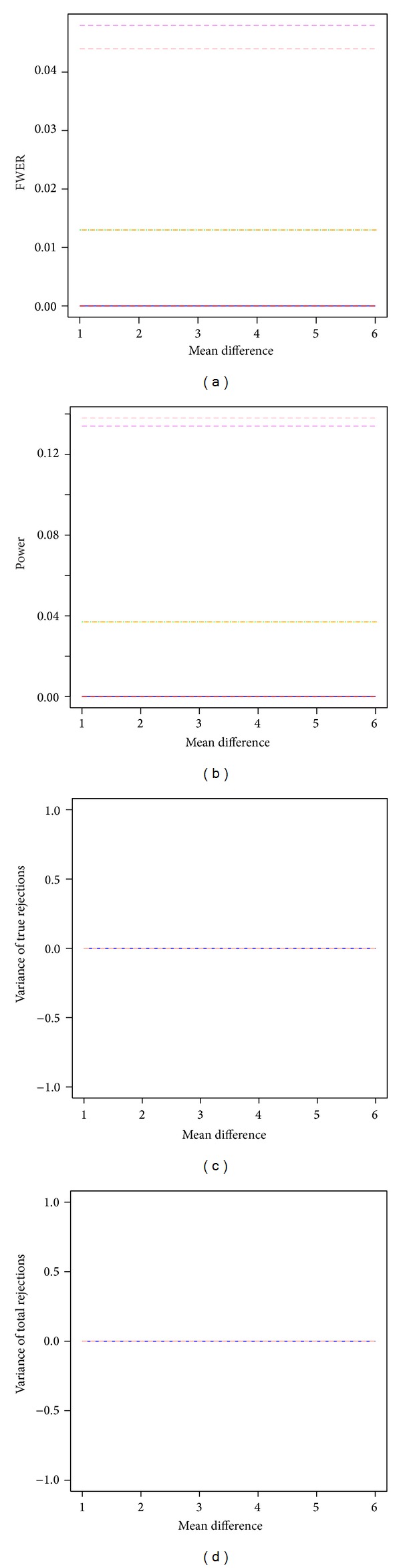
Power and stability properties of resampling-based multiple testing procedures for independent test statistics with FWER controlled at 5% and small sample size of 3 in each group (*m*_0_/*m* = 50%). Solid blue line: permutation single-step max*T* procedure (mt.max*T* function); red dashed line: permutation single-step min*P* (mt.min*P* function); green dotted line: bootstrap single-step max*T* (MTP ss.max*T* function); violet dashed line: bootstrap single-step min*P* (MTP ss.min*P* function); orange dashed line: bootstrap step-down max*T* (MTP sd.max*T* function); pink dashed line: bootstrap step-down min*P* (MTP sd.min*P* function).

For independent test statistics with FWER controlled at 5%, the two bootstrap min*P* procedures outperformed all other tested procedures when sample size is 3 in each group (Figure 1). Both the bootstrap single-step min*P* and the bootstrap step-down min*P* procedures were more powerful than all other tested procedures, and their FWER estimates were close to 5% nominal level. The two permutation-based procedures (mt.max*T* and mt.min*P*) had no power to detect any significant difference between groups, and their FWER estimates were close to 0. The power of the bootstrap max*T* procedures (ss.max*T* and sd.max*T*) were between the permutation procedures and the bootstrap min*P* procedures. The estimated variances of true discoveries and total number of discoveries were around 0 for all tested resampling-based multiple testing procedures. The estimated FWER, power, and stability were constant across effect sizes.

**Figure 2:**
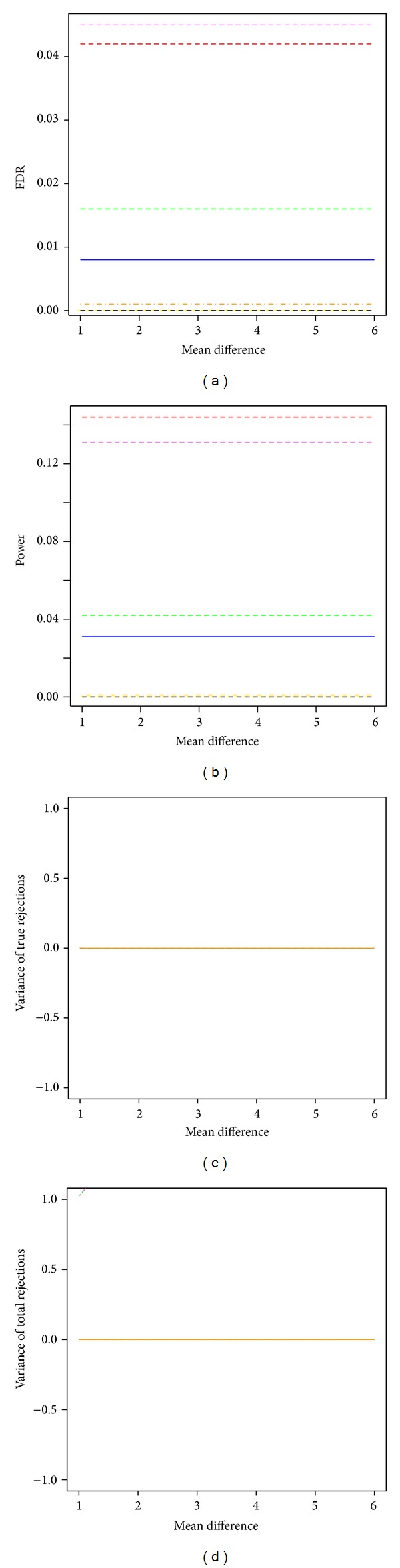
Power and stability properties of resampling-based multiple testing procedures for independent test statistics with FDR controlled at 5% and small sample size of 3 in each group (*m*_0_/*m* = 50%). Yellow dashed line: permutation single-step max*T* procedure (mt.max*T* function); black dashed line: permutation single-step min*P* (mt.min*P* function); solid blue line: bootstrap single-step max*T* (MTP ss.max*T* function); red dashed line: bootstrap single-step min*P* (MTP ss.min*P* function); green dashed line: bootstrap step-down max*T* (MTP sd.max*T* function); violet dashed line: bootstrap step-down min*P* (MTP sd.min*P* function); orange dashed line: SAM procedure (sam function).

The bootstrap single-step and step-down min*P* procedures remained to have the largest power among all tested procedures when FDR was controlled at 5% and sample size was 3 in each group (Figure 2). The FDR estimates from the bootstrap single-step and step-down min*P* procedures also stayed around 5% nominal level. Both the SAM procedure and the two permutation-based max*T* and min*P* procedures had no power to detect any significant difference, and their FDR estimates were also close to 0. Both the FDR estimates and power of the two bootstrap single-step and step-down max*T* procedures were between the SAM procedure, the permutation procedures, and the bootstrap min*P* procedures. All resampling-based multiple testing procedures had estimated variances of true discoveries and total number of discoveries around 0. The estimated FDR, power, and stability were constant across effect sizes.

**Figure 3:**
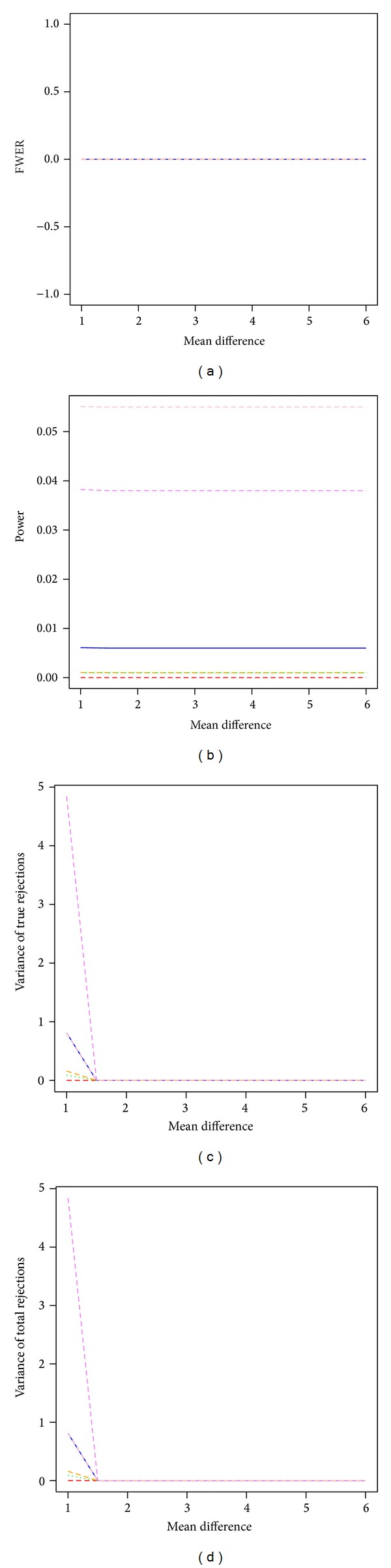
Power and stability properties of resampling-based multiple testing procedures for independent test statistics with FWER controlled at 5% and moderate sample size of 12 in each group (*m*_0_/*m* = 50%). Solid blue line: permutation single-step max*T* procedure (mt.max*T* function); red dashed line: permutation single-step min*P* (mt.min*P* function); green dotted line: bootstrap single-step max*T* (MTP ss.max*T* function); violet dashed line: bootstrap single-step min*P* (MTP ss.min*P* function); orange dashed line: bootstrap step-down max*T* (MTP sd.max*T* function); pink dashed line: bootstrap step-down min*P* (MTP sd.min*P* function).

The bootstrap step-down min*P* procedure had the largest power across all tested procedures when sample size increased to 12 in each group (Figure 3). The bootstrap single-step max*T* procedure, the bootstrap step-down max*T* procedure, and the permutation single-step min*P* procedure showed almost zero power for detecting any difference between groups. All tested procedures had FWER estimates around 0 and showed very small estimated variances of true rejections and variances of total rejections. The estimated FWER and power remained constant across effect sizes.

**Figure 4:**
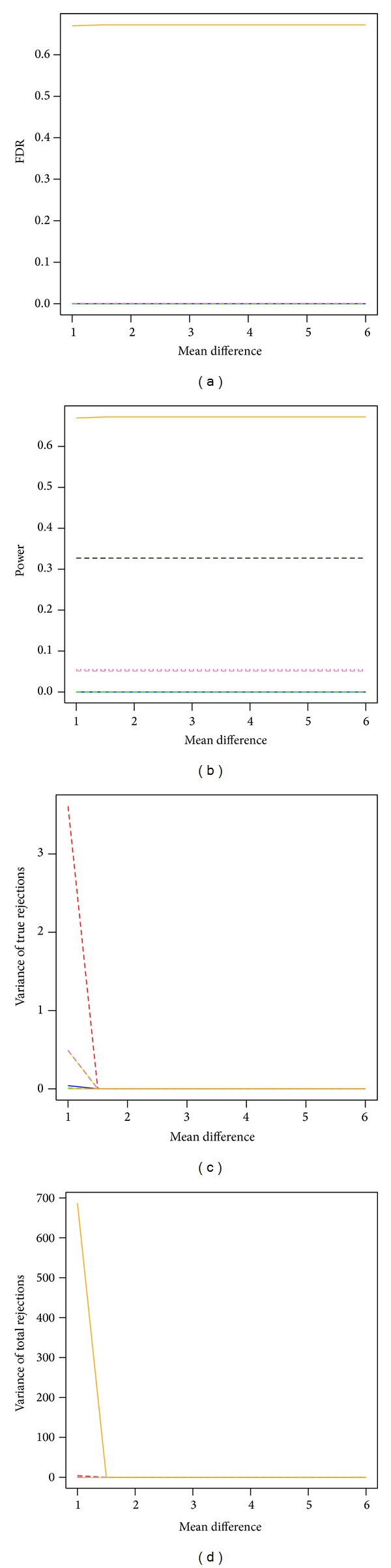
Power and stability properties of resampling-based multiple testing procedures for independent test statistics with FDR controlled at 5% and small sample size of 12 in each group (*m*_0_/*m* = 50%). Yellow dashed line: permutation single-step max*T* procedure (mt.max*T* function); black dashed line: permutation single-step min*P* (mt.min*P* function); solid blue line: bootstrap single-step max*T* (MTP ss.max*T* function); red dashed line: bootstrap single-step min*P* (MTP ss.min*P* function); green dashed line: bootstrap step-down max*T* (MTP sd.max*T* function); violet dashed line: bootstrap step-down min*P* (MTP sd.min*P* function); orange dashed line: SAM procedure (sam function).

The permutation single-step max*T* procedure and the permutation single-step min*P* procedure performed the best when FDR is controlled at 5% and sample size is 12 in each group (Figure 4). The two permutation max*T* and min*P* procedures had much larger power than the four bootstrap MTP procedures and also had estimated FDR less than 5%. The SAM procedure failed to control the FDR at the desired level of 5%, although it had larger power than all other tested procedures. The estimated variances of total discoveries from the SAM procedure were much larger than all other procedures when the effect size is around 1. The permutation single-step max*T* and min*P* procedures had small variances of true discoveries and total discoveries. The four bootstrap MTP procedures had low power but similar stability as the permutation max*T* and min*P* procedures. The estimated FDR and power were also constant across effect sizes.

### 3.2. Simulation Results for Dependent Test Statistics

**Figure 5:**
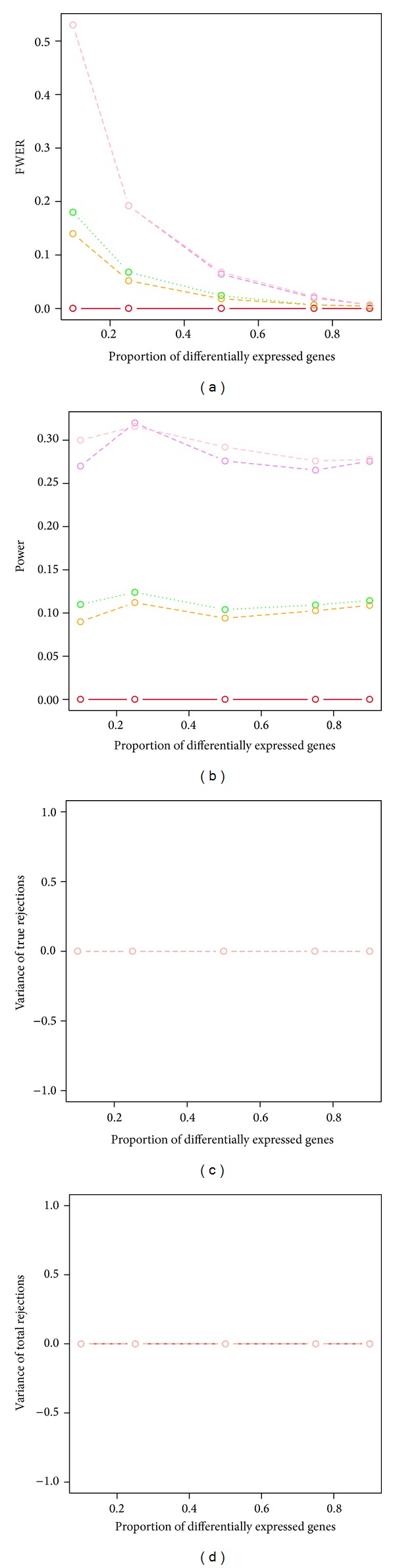
Power and stability properties of resampling-based multiple testing procedures for dependent test statistics with random correlations and the FWER is controlled at 5% (*n* = 3 in each group). Blue dashed line: permutation single-step max*T* procedure (mt.max*T* function); solid red line: permutation single-step min*P* (mt.min*P* function); green dotted line: bootstrap single-step max*T* (MTP ss.max*T* function); orange dashed line: bootstrap step-down max*T* (MTP sd.max*T* function); violet dashed line: bootstrap single-step min*P* (MTP ss.min*P* function); pink dashed line: bootstrap step-down min*P* (MTP sd.min*P* function).

The two bootstrap min*P* procedures (ss.min*P* and sd.min*P*) showed higher power than all other tested procedures across various proportions of nontrue null hypotheses, when test statistics are dependent and FWER is controlled. The two bootstrap min*P* procedures had desired FWER control as well, when the proportions of nontrue null hypotheses were greater than 50% (Table 1 and Figure 5). The two bootstrap max*T* procedures (ss.max*T* and sd.max*T*) had lower power than the two bootstrap min*P* procedures. They showed desired FWER control, however, when the proportions of nontrue null hypotheses were over 25%. The permutation single-step max*T* and min*P* procedures had no power to detect any significant difference between groups. All resampling-based procedures had estimated variances of true discoveries and total discoveries around 0 across various proportions of nontrue null hypotheses, when sample size was as small as 3 in each group.

**Figure 6:**
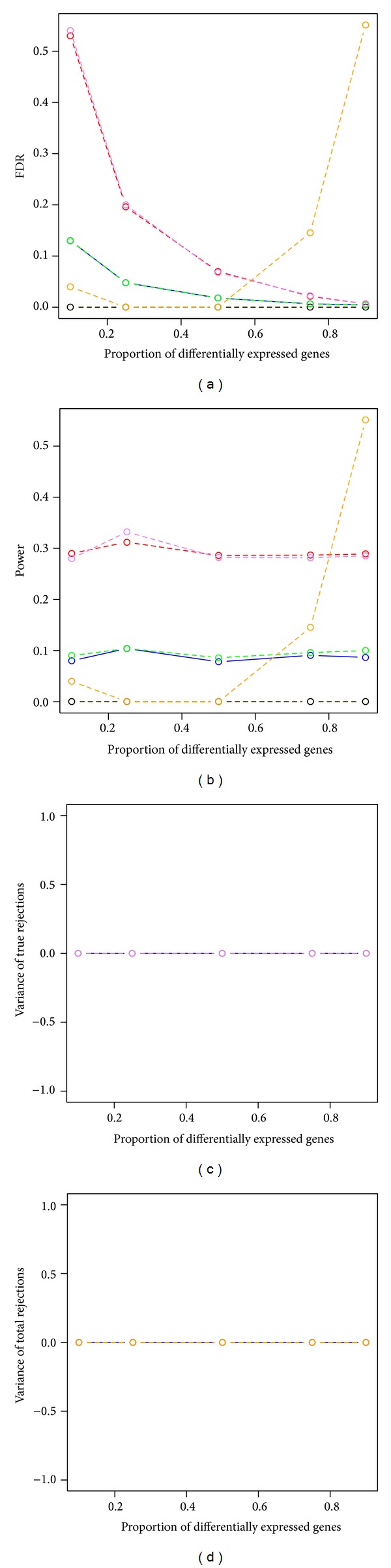
Power and stability properties of resampling-based multiple testing procedures for dependent test statistics with random correlations and the FDR is controlled at 5% (*n* = 3 in each group). Yellow dashed line: permutation single-step max*T* procedure (mt.max*T* function); black dashed line: permutation single-step min*P* (mt.min*P* function); solid blue line: bootstrap single-step max*T* (MTP ss.max*T* function); green dashed line: bootstrap step-down max*T* (MTP sd.max*T* function); red dashed line: bootstrap single-step min*P* (MTP ss.min*P* function); violet dashed line: bootstrap step-down min*P* (MTP sd.min*P* function); orange dashed line: SAM procedure (sam function).

The power and stability of all four bootstrap methods (ss.min*P*, sd.min*P*, ss.max*T*, and sd.max*T*) and the two permutation methods (mt.max*T* and mt.min*P*) showed similar results, when FDR were controlled, as that when FWER were controlled (Table 2 and Figure 6). The SAM procedure had decent FDR control, but very low power when the proportions of nontrue null hypotheses were less than 50%. Both the estimated FDR and power increased when the proportions of nontrue null hypotheses were greater than 50% for the SAM procedure.

### 3.3. Real Data Example

The gene expression levels of 12625 genes from 6 subjects on log2 scale were used to compare total number of discoveries identified from all tested resampling-based multiple testing procedures (Table 3). The two bootstrap min*P* procedures had more rejections than the two bootstrap max*T* procedures, when FWER was controlled at 5%. The bootstrap step-down min*P* and single-step min*P* procedures remained higher number of rejections than the bootstrap step-down max*T* and single-step max*T* procedures, when FDR was controlled at 5%. The SAM procedure only rejected 2 genes. The permutation max*T* and min*P* procedures rejected none of those genes. The bootstrap multiple testing procedures has higher power than all other tested procedures and rejected much more null hypotheses compared to the permutation test procedures. The bootstrap min*P* procedures rejected more hypotheses than the bootstrap max*T* procedures. The total number of rejections from this real microarray data analysis is consistent with the results from the simulation studies.

## 4. Discussion

This paper investigated the power and stability properties of several popular resampling-based multiple testing procedures for both independent and dependent test statistics, when sample size is small or moderate, using available functions in *R*. Our simulation results and real data example show that the bootstrap single-step and step-down min*P* procedures perform the best for both small sample size data (3 in each group) and moderate sample size data (12 in each group) when FWER control is desired. The bootstrap single-step and step-down min*P* procedures are the best when FDR control is desired for data with small sample size (3 in each group). The permutation max*T* and min*P* procedures perform the best for data with moderate sample size when FDR control is desired. The SAM procedure overestimates FDR, although it has higher power than the permutation and bootstrap max*T* and min*P* procedures.

The simulation results also showed that the permutation test procedures have no power to detect any significant differences between groups when sample size is as small as 3 in each group; the permutation test procedures perform well when sample size increases to 12 in each group; the SAM procedure has no power for detecting significant differences when the proportion of nontrue null hypotheses is less than 50% and sample size is 3; the bootstrap multiple testing procedures perform better than the permutation test procedures and the SAM procedure for small sample size data.

The zero power of the permutation test procedures is due to its limited number of permutated test statistics for data set with small sample sizes. For example, the complete number of enumeration is only 20 for both permutation single-step max*T* procedure and permutation single-step min*P* procedure when sample size is only 3 in each group. Thus, the smallest raw *P*  value from the permutation procedures will be 0.05. After adjusting the raw *P*  values to control FWER or FDR, all adjusted *P*  values will be larger than 0.05, thus no hypotheses will be rejected. As such, the estimated FWER, FDR, and power will all be zero.

Our current investigation only focuses on normally distributed data. Further investigation is needed to extend the distribution in the simulations from multivariate normal to other distributions, such as lognormal and binomial distributions. To examine the power and stability properties of those resampling-based multiple testing procedures, under nonnormal distributions, will be a focus for our future research.

## Figures and Tables

**Table 1:** Comparison of the estimated FWER and power for the resampling-based multiple testing procedures with FWER controlled at 5%.

	(*m* − *m*_0_)/*m*	mt.max*T *	mt.min*P *	ss.max*T *	ss.min*P *	sd.max*T *	sd.min*P *
FWER	0.10	0.00	0.00	0.18	0.53	0.14	0.53
	0.25	0.00	0.00	0.07	0.19	0.05	0.19
	0.50	0.00	0.00	0.02	0.06	0.02	0.07
	0.75	0.00	0.00	0.01	0.02	0.01	0.02
	0.90	0.00	0.00	0.00	0.01	0.00	0.01

Power	0.10	0.00	0.00	0.11	0.27	0.09	0.30
	0.25	0.00	0.00	0.12	0.32	0.11	0.32
	0.50	0.00	0.00	0.10	0.28	0.09	0.29
	0.75	0.00	0.00	0.11	0.27	0.10	0.28
	0.90	0.00	0.00	0.11	0.28	0.11	0.28

mt.max*T*: permutation single-step max*T* procedure; mt.min*P*: permutation single-step min*P* procedure; ss.max*T*: bootstrap single-step max*T* procedure; ss.min*P*: bootstrap single-step min*P* procedure; sd.max*T*: bootstrap step-down max*T* procedure; sd.min*P*: bootstrap step-down min*P* procedure.

**Table 2:** Comparison of the estimated FDR and power for the resampling-based multiple testing procedures with FDR controlled at 5%.

	(*m* − *m*_0_)/*m*	mt.max*T *	mt.min*P *	ss.max*T *	ss.min*P *	sd.max*T *	sd.min*P *	sam
FDR	0.10	0.00	0.00	0.13	0.53	0.13	0.54	0.04
	0.25	0.00	0.00	0.05	0.20	0.05	0.20	0.00
	0.50	0.00	0.00	0.02	0.07	0.02	0.07	0.00
	0.75	0.00	0.00	0.01	0.02	0.01	0.02	0.15
	0.90	0.00	0.00	0.00	0.01	0.00	0.01	0.55

Power	0.10	0.00	0.00	0.08	0.29	0.09	0.28	0.04
	0.25	0.00	0.00	0.10	0.31	0.10	0.33	0.00
	0.50	0.00	0.00	0.08	0.29	0.09	0.28	0.00
	0.75	0.00	0.00	0.09	0.29	0.10	0.28	0.15
	0.90	0.00	0.00	0.09	0.29	0.10	0.29	0.55

mt.max*T*: permutation single-step max*T* procedure; mt.min*P*: permutation single-step min*P* procedure; ss.max*T*: bootstrap single-step max*T* procedure; ss.min*P*: bootstrap single-step min*P* procedure; sd.max*T*: bootstrap step-down max*T* procedure; sd.min*P*: bootstrap step-down min*P* procedure; sam: the SAM procedure.

**Table 3:** Comparisons of number of total discoveries for the resampling-based multiple testing procedures for the ovarian cancer example with 12,625 genes.

Resampling methods	Rejected number of hypothesis	
	FWER controlled at 5%	FDR controlled at 5%
mt.max*T *	0	0
mt.min*P *	0	0
ss.max*T *	250	385
sd.max*T *	407	397
ss.min*P *	1785	1649
sd.min*P *	1766	1706
sam		2

mt.max*T*: permutation single-step max*T* procedure; mt.min*P*: permutation single-step min*P* procedure; ss.max*T*: bootstrap single-step max*T* procedure; ss.min*P*: bootstrap single-step min*P* procedure; sd.max*T*: bootstrap step-down max*T* procedure; sd.min*P*: bootstrap step-down min*P* procedure; sam: the SAM procedure.
